# Excess Mortality and Long-Term Disability from Healthcare-Associated *Staphylococcus aureus* Infections: A Population-Based Matched Cohort Study

**DOI:** 10.1371/journal.pone.0071055

**Published:** 2013-08-06

**Authors:** Chiu-Hsia Su, Shan-Chwen Chang, Jer-Jea Yan, Shu-Hui Tseng, Li-Jung Chien, Chi-Tai Fang

**Affiliations:** 1 Institute of Epidemiology and Preventive Medicine, College of Public Health, National Taiwan University, Taipei, Taiwan; 2 Fifth division, Centers for Disease Control, Taipei, Taiwan; 3 Department of Internal Medicine, National Taiwan University Hospital, Taipei, Taiwan; The University of Hong Kong, China

## Abstract

**Background:**

*Staphylococcus aureus* is a leading cause of healthcare-associated infections (HAIs), but the impact of *S. aureus* HAIs on the long-term survival and functional status of hospitalized patients remain unknown. This study aimed to examine whether *S. aureus* HAIs increase the risks for long-term mortality and disability.

**Methods:**

We conducted a retrospective population-based matched cohort study of inpatients at 8 medical centers, 43 regional hospitals, and 63 local hospitals which participated in the Taiwan Nosocomial Infection Surveillance (TNIS). We individually matched 3070 patients with *S. aureus* HAIs to 6140 inpatients without HAIs at a 1∶2 ratio by age, gender, hospital, specialty, underlying diseases, and the length of stay before onset of the *S. aureus* HAI. Main outcome measures are one-year excess risks for mortality, new-onset chronic ventilator dependence, and new-onset dialysis-dependent end-stage renal disease.

**Results:**

We found that patients with *S. aureus* HAIs had an excess one-year mortality of 20.2% compared with matched uninfected inpatients (*P*<0.001). The excess risk for new-onset chronic ventilator dependence and dialysis-dependent end-stage renal disease was 7.3% and 2.6%, respectively (*P*s<0.001). *S. aureus* HAIs were also associated with an excess hospital stay of 12 days and an extra cost of $5978 (*P*s<0.001).

**Conclusion:**

*S. aureus* HAIs have substantial negative effect on the long-term outcome of hospitalized patients in terms of both mortality and disability, which should be taken into consideration in future cost-effectiveness studies of the control and prevention interventions for *S. aureus* HAIs.

## Introduction

*Staphylococcus aureus* is a leading cause of healthcare-associated infections (HAIs) [1], [2]. *S. aureus* infections can cause severe sepsis complicated by acute renal failure and respiratory failure requiring intensive care [3], [4]. *S. aureus* bacteremia is associated with an in-hospital mortality of as high as 15–60% [5], especially in critically ill patients [6]–[8]. Bacteremia of methicillin-resistant *S. aureus* (MRSA) [9]–[13] has a higher attributable mortality than that of methicillin-susceptible *S. aureus* (MSSA) [2], [14]. Thus, *S. aureus* HAIs can have substantial impacts on the patient’s survival and well-being.

The negative effects of *S. aureus* HAIs on the outcomes of hospitalized patients have not yet been well studied. The existing literature includes only six small studies, which reported an increased risk for short-term mortality by 2.2–7.3 folds in patients with *S. aureus* HAIs compared to inpatients without HAIs [15]–[20]. Most studies focused on surgical site infection (sample size: 18–286 cases) [15]–[17] or bloodstream infection (sample size: 19 cases) [18]; only one study examined all-type *S. aureus* HAIs (sample size: 27 cases) [19]. None of the studies have investigated the impact of *S. aureus* HAIs on mortality beyond 90 days [21]. The functional status of survivors has not been studied, either. The acute respiratory or renal failure occurring during sepsis may be irreversible and thus result in long-term ventilator or dialysis dependence, causing huge financial burdens.

Taiwan Nosocomial Infection Surveillance (TNIS) is a nationwide surveillance system that collects HAI data from more than 100 participating hospitals throughout Taiwan. The TNIS data show that *S. aureus* is one of the leading causative pathogens of HAIs in Taiwan [22]. MRSA accounts for up to 79% and 81% of all *S. aureus* isolates at regional hospitals and medical centers, respectively [22]. To understand the impact of *S. aureus* HAIs on the long-term outcomes of hospitalized patients, we conducted a nationwide population-based matched cohort study of inpatients at 114 hospitals participating in the TNIS.

## Methods

### Study Design

We conducted a retrospective population-based matched cohort study comparing outcomes between hospitalized patients with *S. aureus* HAIs and patients without HAIs, matched by age, gender, hospital, specialty, underlying diseases, and the length of stay before onset of the *S. aureus* HAI. The main outcomes were one-year excess risks for mortality, new-onset chronic ventilator dependence, and new-onset dialysis-dependent end-stage renal disease.

### Ethical Statement

To protect the privacy of the patients, the personal identification numbers were encrypted before database linking. The study protocol (no. TwCDCIRB990008) was reviewed and approved a priori by the institutional review board (IRB) of Taiwan Centers for Diseases Control (Taipei, Taiwan). The IRB approved the exemption of informed consent because all personal information had been anonymized.

### Settings

Taiwan Centers for Diseases Control established the TNIS in 2006 and invited all hospitals to voluntarily participate. By 2008, 114 out of the total 495 hospitals in Taiwan had joined the TNIS and notified their HAIs cases with registration of the patients’ personal identification (ID) numbers. The 114 hospitals included 8 medical centers, 43 regional hospitals, and 63 local hospitals (with a median bed capacity of 1318, 581, and 182, respectively), which had a total of 3307878 hospitalizations covered by the NHI during the study period from 2006 through 2008.

### HAI Surveillance and Notification

In all participating hospitals, infection control nurses routinely review all hospitalizations for all types of HAIs (including bloodstream infection, pneumonia, surgical site infection, urinary tract infection, and other types of HAIs) using the US Centers for Disease Control and Prevention (CDC) (Atlanta, GA, USA) surveillance definitions [23]. The identified HAI cases were notified to the TNIS. The reported data included the patient’s age, gender, HAI onset date, site of infection, and microbiological results (e.g. organisms isolated from blood, urine, respiratory tract, surgical sites, and other non-sterile sites, as well as antimicrobial susceptibility). The onset date was the date when the first clinical symptom(s)/sign(s) occurred or the earliest positive culture was sampled, as specified for the type of HAI by the CDC definition.

### Patients with *S. aureus* HAIs

**Figure 1:**
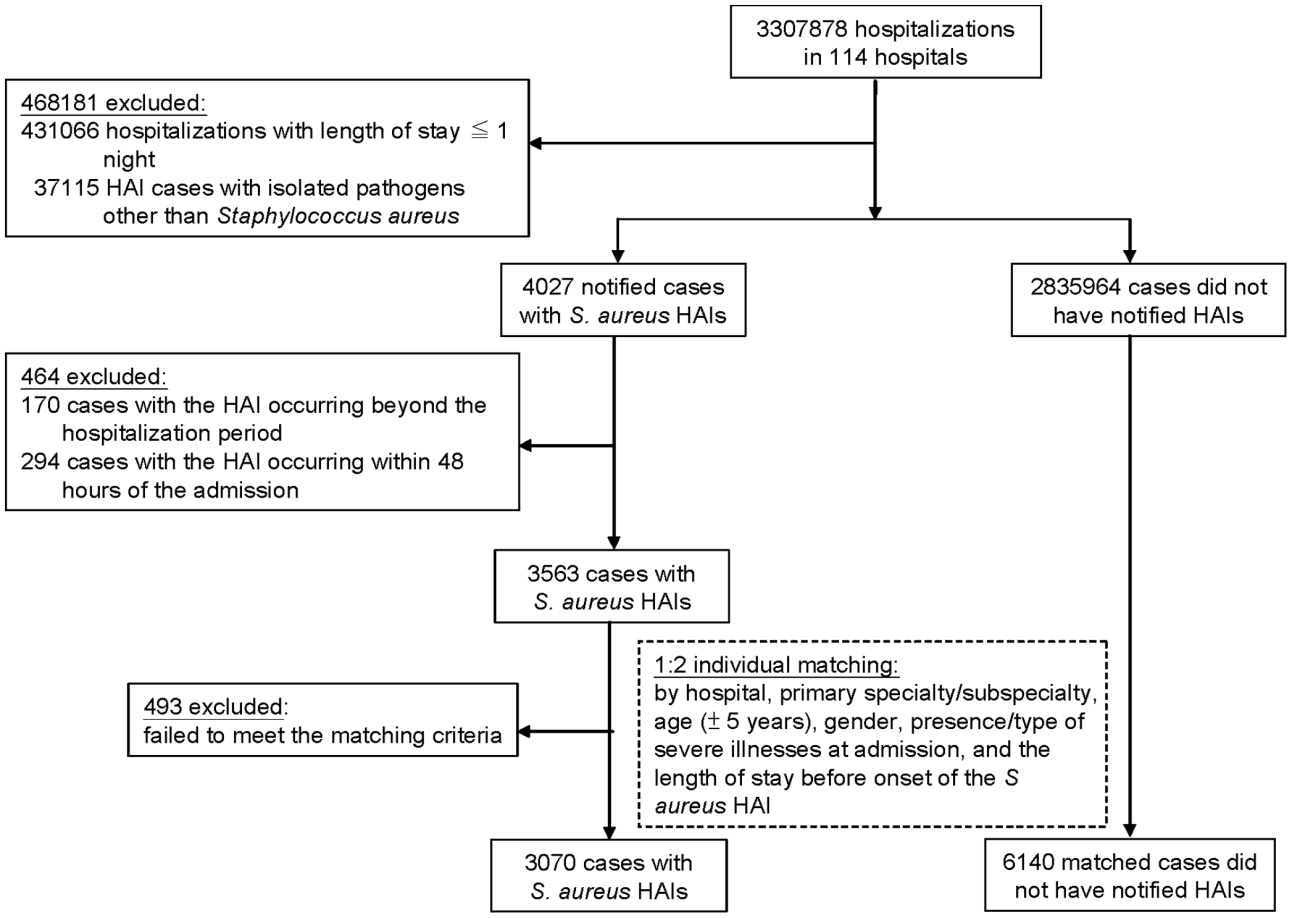
Flowchart of patient selection for matching.

We included all notified *S. aureus* HAIs cases that occurred at least 48 hours after admission in 2006–2008 for linkage with the NHI dataset. The flowchart is shown in Figure 1. If a patient had multiple episodes of HAIs during hospitalization, only the first episode and its first isolate were considered in this study. Cases with the HAI occurring within 48 hours of the admission or beyond the hospitalization period were excluded, because we used the length of stay before onset of the *S. aureus* HAI as one of the matching variables to identify a suitable matched uninfected inpatient [8], [14].

### Matched Inpatients without HAIs

Each *S. aureus* HAI case was individually matched at a 1∶2 ratio to inpatients without HAIs that were hospitalized during the same study period. The matching was based on age (within a 5-year difference), gender, as well as the same hospital, primary specialty/subspecialty, and indicators of underlying disease severity–including the length of stay before onset of the *S. aureus* HAI [8], [14] and the presence and type of seven classes of severe illnesses at admission (i.e. cancer, dialysis-dependent end stage renal disease, liver cirrhosis with complications, chronic ventilator dependence, major trauma, generalized autoimmune syndrome, and spinal injury/myeleterosis). If there were more than two candidate uninfected inpatients, we chose the two that had the closest admission dates to that of the *S. aureus* HAI case. If no suitable match was found, we reduced the matching requirement of primary specialty/subspecialty to just primary specialty. If a suitable match still could not be identified, we considered the matching process to have failed.

We used the NHI database to obtain patient data for individual matching and validation of comparability. The NHI in Taiwan has a coverage rate of 99% due to universal health insurance [24]. The NHI claims data recorded five major diagnoses (i.e. one primary diagnosis and up to four secondary diagnoses) for the patient, which were reported by the hospital based on the ICD–9–CM coding system. We ascertained the presence and type of severe illnesses using the Catastrophic Illness Registry, which is a subset of the NHI database (and thus has the same coverage rate). There are 30 major categories of catastrophic illnesses for which patient copayment can be exempted. The certification, which is strictly regulated by the NHI bureau, requires independent evaluation by two specialist physicians to confirm both the diagnosis and irreversibility of the illness [25].

### Validation of Comparability

To validate comparability between the *S. aureus* HAI cases and matched uninfected inpatients on baseline characteristics before onset of *S. aureus* HAIs, we examined the between-group difference on clinical variables unrelated to HAIs (i.e. the presence of ischemic heart disease, congestive heart failure, stroke, diabetes, hypertension, elective surgical procedures, and medications for treating cardiovascular and/or neoplastic disorders).

### Ascertainment of Outcomes

We derived the data on survival status and date of death using the National Death Registry (from Department of Health, Taiwan), which contains all the death certificates of Taiwanese citizens. The data on new-onset chronic ventilator dependence and dialysis-dependent end-stage renal disease were ascertained using the Catastrophic Illness Registry. To ensure a 100% one-year follow-up rate, data of both registries were updated to the end of year 2009. We used the date of Catastrophic Illness Certificate application as the onset date of chronic ventilator dependence and dialysis-dependent end-stage renal disease. To distinguish old events that were already present at admission from new-onset events that occurred after the index date, we defined the index date for *S. aureus* HAI patients as the onset date of the *S. aureus* HAI; that for uninfected patients was the admission date plus the length of stay before onset of the *S. aureus* HAI of the matched case. We used three linkage variables (encrypted personal ID, encrypted hospital ID, and admission date) to link the anonymized patient data between different datasets.

The data of hospital costs were obtained from the NHI dataset, which recorded the total cost (including diagnosis, laboratory, drug, ward, therapeutic-procedure, and special-material fees) for the entire hospitalization period of each patient.

### Statistical Analysis

We compared the main outcomes between the *S. aureus* HAI group and the uninfected group using multivariate conditional logistic regression stratified by matched pairs, with adjustment for the effects of diabetes mellitus and hypertension. We compared the length of hospital stay and the hospital cost between two groups using the random effect model. All statistical analyses were performed using SAS, version 9.2 (SAS Institute Inc., Cary, NC, USA). Statistical significance of P values was interpreted with Bonferroni’s correction for multiple comparisons.

## Results

The 114 hospitals reported a total of 47729 HAI cases during 2006–2008. Linking between the TNIS and the NHI dataset failed for 6587 cases (13.8%) due to inconsistency in one or more of the three linkage variables. Among the remaining 41142 HAI cases, the isolated pathogen was *S. aureus* for 4027 cases. Of them, 3563 cases met the inclusion criteria and 3070 cases were successfully matched to 6140 inpatients without HAIs (successful matching rate 86.2% [3070/3563]) (Figure 1). Compared to *S. aureus* HAI cases with successful matching, the *S. aureus* HAI cases with unsuccessful matching (n = 493) had a longer average length of stay before onset of the *S. aureus* HAI (95 vs. 20 days) and were more likely to have a severe illness at admission (14.0% vs. 3.7% for dialysis-dependent end-stage renal disease; 21.1% vs. 2.0% for chronic ventilator dependence) (all *P*s <0.001).

Of the 3070 *S. aureus* HAI cases, the causal *S. aureus* strains were MRSA in 2201 cases (71.7%). Patients with MRSA HAIs tended to be older (mean age: 68 vs. 62 years), had a longer average length of stay before onset of the HAI (23 vs. 13 days), and were more likely to have a severe illness at admission (4.0% vs. 2.9% for dialysis-dependent end-stage renal disease; 2.5% vs. 0.6% for chronic ventilator dependence), compared with patients with MSSA HAIs (all *P*s <0.001).

The baseline characteristics of the 3070 matched pairs are shown in Table 1. There was no statistically significant between-group difference in the matching variables and the comparability-validation variables, with the only exceptions of diabetes mellitus and hypertension. Compared with the *S. aureus* HAI group, the uninfected group had a slightly higher proportion of patients with diabetes mellitus (22.1% vs. 19.4%, *P*<0.001) and hypertension (23.2% vs. 16.8%, *P*<0.001), as well as a lower average number of diagnoses recorded in the NHI dataset (4.3 vs. 4.7, *P*<0.001).

**Table 1:** Baseline characteristics of 3070 matched pairs.

	*S. aureus* HAI Patients (n = 3070)	Matched Patients without HAIs (n = 6140)	*P* value
**Matching Variables**			
Age, mean±SD/median (IQR)	67±19/72 (56–80)	67±19/72 (56–80)	–
Gender, female (%)	1051 (34.2)	2108 (34.2)	–
Type of hospital, n (%)			
Medical center	944 (30.8)	1888 (30.8)	–
Regional hospital	1610 (52.4)	3220 (52.4)	–
Local hospital	516 (16.8)	1032 (16.8)	–
Primary specialty,* n (%)			
Neurosurgery	259 (8.4)	518 (8.4)	–
Medicine	236 (7.7)	472 (7.7)	–
Surgery	170 (5.5)	345 (5.6)	–
Neurology	136 (4.4)	272 (4.4)	–
Orthopedics	116 (3.8)	232 (3.8)	–
Pediatrics	70 (2.3)	136 (2.2)	–
Plastic Surgery	61 (2.0)	122 (2.0)	–
Family Medicine	48 (1.6)	96 (1.6)	–
Severe illness, n (%)			
Cancer	520 (17.0)	1040 (17.0)	–
dialysis-dependent End-stage renal disease	114 (3.7)	228 (3.7)	–
Liver cirrhosis with complications	60 (2.0)	120 (2.0)	–
Chronic ventilator dependence	60 (2.0)	120 (2.0)	–
Generalized autoimmune syndrome	32 (0.5)	16 (0.5)	–
Spinal injury/myeleterosis	6 (0.2)	12 (0.2)	–
Major trauma	14 (0.5)	28 (0.5)	–
**Validation Variables**			
Diagnosis, n (%)			
Ischemic heart disease	217 (7.1)	480 (7.8)	0.15
Congestive heart failure	226 (7.4)	444 (7.2)	0.81
Stroke	424 (13.8)	848 (13.8)	1.0
Diabetes mellitus	594 (19.4)	1354 (22.1)	0.001†
Hypertension	516 (16.8)	1427 (23.2)	<0.001†
Procedure, n (%)			
Total joint replacement	16 (0.5)	43 (0.7)	0.31
Coronary artery bypass graft	33 (1.1)	43 (0.7)	0.06
Rectoscopy	11 (0.4)	13 (0.2)	0.19
Laparoscopy	6 (0.2)	14 (0.2)	0.52
Medication, n (%)			
Statins	154 (5.0)	310 (5.0)	0.95
Streptokinase	17 (0.6)	25 (0.4)	0.33
Antigout preparations	293 (9.5)	506 (8.2)	0.04
Antineoplastic agents	159 (5.2)	387 (6.3)	0.03

Abbreviations: HAI, healthcare-associated infection; SD, standard deviation; IQR, interquartile range.

* Eight out of 15 primary specialties with the most patients were listed.

† Statistically significant, after Bonferroni correction (*P*<0.05/13 = 0.0038).

**Figure 2:**
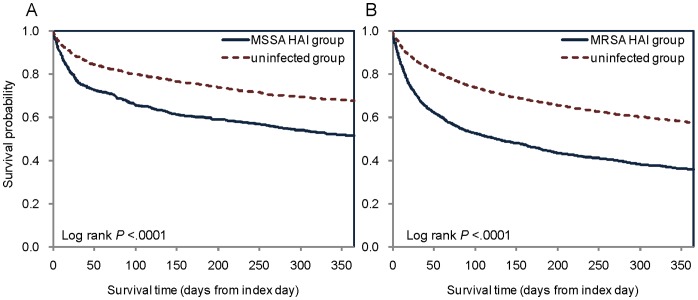
Kaplan-Meier survival curves (A) MSSA patients (n = 869) and their matched uninfected patients (n = 1738). (B) MRSA patients (n = 2201) and their matched uninfected patients (n = 4402).

Table 2 summarizes the main outcomes. *S. aureus* HAI cases had an excess in-hospital mortality, mortality within 30 days after discharge, and one-year mortality of 19.9%, 21.1%, and 20.2%, respectively (all *P*s <0.001) (Table 2). The excess one-year mortality was highest for nosocomial pneumonia (28.5%) and bloodstream infection (22.3%) (Table 3). MRSA and MSSA cases had an excess one-year mortality of 21.8% and 16.1%, respectively (Table 3 and Figure 2). *S. aureus* HAIs cases also had an excess risk for new-onset chronic ventilator dependence during hospitalization, within 30 days after discharge, and within one-year (6.8%, 7.6%, and 7.3%, respectively, all *P*s <0.001). The excess risk for new-onset dialysis-dependent end-stage renal disease during hospitalization, within 30 days after discharge, and within one-year was 1.7%, 2.3%, and 2.6%, respectively (all *P*s <0.001). After adjusting for the presence of diabetes mellitus and hypertension, the differences in outcomes between the *S. aureus* HAI group and the uninfected group remained highly statistically significant (all *P*s <0.001) (Table 2).

**Table 2:** Excess risks for mortality and new-onset organ failure in patients with *S. aureus* HAIs.

Outcomes	Endpoint of Observation*	*S. aureus* HAI Patients	Matched Patients without HAIs	Excess Risk(%)	OR	Adjusted OR‡
Mortality	Number at risk#	3070	6140	–	–	–
	Discharge	956 (31.1)	691 (11.3)	19.9	4.5†	4.3†
	30-day after discharge	1188 (38.7)	1082 (17.6)	21.1	3.8†	3.7†
	one-year	1828 (59.5)	2416 (39.3)	20.2	3.2†	3.1†
Chronic ventilator dependence	Number at risk#	3010	6020	–	–	–
	Discharge	279 (9.3)	151 (2.5)	6.8	4.8†	4.6†
	30-day after discharge	329 (10.9)	203 (3.4)	7.6	4.2†	4.1†
	one-year	393 (13.1)	349 (5.8)	7.3	2.8†	2.7†
Dialysis-dependent end-stage	Number at risk#	2956	5912	–	–	–
renal disease	Discharge	77 (2.6)	53 (0.9)	1.7	3.5†	4.1†
	30-day after discharge	120 (4.1)	105 (1.8)	2.3	2.9†	3.6†
	one-year	153 (5.2)	153 (2.6)	2.6	2.6†	3.2†

Abbreviations: HAI, healthcare-associated infection; OR, odds ratio.

* Follow-up duration from index date to endpoint of observation.

# Number at risk: the number of patients who have not yet developed the outcomes at admission.

‡ Adjusted for diabetes mellitus and hypertension.

† Statistically significant, after Bonferroni correction (all *P*<0.05/18 = 0.0028).

**Table 3:** Subgroup analysis of excess one-year mortality.

	*S. aureus* HAI Patients		Matched Patients without HAIs		% Difference	*P* value
Variables	n	Event (%)	n	Event (%)		
one-year mortality, n (%)	3070	1828 (59.5)	6140	2416 (39.3)	20.2	<0.001†
By site of infection of index *S. aureus* HAI cases						
Bloodstream infection	1329	878 (66.1)	2658	1162 (43.7)	22.3	<0.001†
Pneumonia	785	540 (68.8)	1570	632 (40.3)	28.5	<0.001†
Urinary tract infection	206	111 (53.9)	412	186 (45.1)	8.7	<0.001†
Surgical site infection	310	102 (32.9)	620	127 (20.5)	12.4	<0.001†
Others	440	197 (44.8)	880	309 (35.1)	9.7	<0.001†
By antimicrobial resistance of index *S. aureus* HAI cases						
MSSA	869	419 (48.2)	1738	558 (32.1)	16.1	<0.001†
MRSA	2201	1409 (64.0)	4402	1858 (42.2)	21.8	<0.001†
By presence of severe illnesses* at admission of index *S. aureus* HAI cases						
No	2295	1255 (54.7)	4590	1491 (32.5)	22.2	<0.001†
Yes	775	573 (73.9)	1550	925 (59.7)	14.2	<0.001†

Abbreviations: HAI, healthcare-associated infection; SD, standard deviation; MSSA, methicillin-susceptible *S. aureus*; MRSA, methicillin- resistant *S. aureus*.

* Any of the 7 classes of severe illnesses (cancer, dialysis-dependent end stage renal disease, liver cirrhosis with complications, chronic ventilator dependence, generalized autoimmune syndrome, spinal injury/myeleterosis, and major trauma).

† Statistically significant, after Bonferroni correction (*P*<0.05/10 = 0.005).

Patients with *S. aureus* HAIs had an excess hospital stay of 12 days and an extra hospital cost of $5978 compared with the matched uninfected patients (Table 4). The differences were significant in subgroup analysis by the site of infection (bloodstream, pneumonia, urinary tract, and surgical site of infection), the type of antimicrobial resistance (MSSA and MRSA), and the presence (or absence) of severe illnesses at admission (all *P*s <0.001) (Table 4).

**Table 4:** Subgroup analysis of excess hospital stay and costs.

	*S. aureus* HAI Patients		Matched Patients without HAIs		Mean Difference	*P* value
Variables	n	Mean (SD)	n	Mean (SD)		
Length of stay, mean (SD), days	3070	45 (51)	6140	33 (50)	12	<0.001†
By site of infection of index *S. aureus* HAI cases						
Bloodstream infection	1329	42 (35)	2658	33 (43)	9	<0.001†
Pneumonia	785	47 (54)	1570	30 (48)	17	<0.001†
Urinary tract infection	206	51 (64)	412	46 (63)	5	<0.001†
Surgical site infection	310	44 (36)	620	28 (28)	16	<0.001†
Others	440	50 (79)	880	38 (73)	12	<0.001†
By antimicrobial resistance of index *S. aureus* HAI cases						
MSSA	869	34 (40)	1738	23 (36)	11	<0.001†
MRSA	2201	50 (54)	4402	37 (54)	13	<0.001†
By presence of severe illnesses* at admission of index*S. aureus* HAI cases						
No	2295	46 (53)	4590	33 (49)	13	<0.001†
Yes	775	42 (45)	1550	35 (54)	7	<0.001†
Cost of hospitalization, mean (SD), in US dollars ‡	3070	12879 (13043)	6140	6900 (9006)	5979	<0.001†
By site of infection of index *S. aureus* HAI cases						
Bloodstream infection	1329	12441 (12822)	2658	7085 (9357)	5355	<0.001†
Pneumonia	785	14657 (13431)	1570	6285 (8374)	8373	<0.001†
Urinary tract infection	206	11468 (12448)	412	8477 (10351)	2991	<0.001†
Surgical site infection	310	12922 (13114)	620	6488 (6680)	6435	<0.001†
Others	440	11658 (12946)	880	6991 (9645)	4667	<0.001†
By antimicrobial resistance of index *S. aureus* HAI cases						
MSSA	869	8280 (8869)	1738	4378 (5520)	3903	<0.001†
MRSA	2201	14694 (13951)	4402	7896 (9880)	6798	<0.001†
By presence of severe illnesses* at admission of index*S. aureus* HAI cases						
No	2295	13437 (13571)	4590	6852 (8826)	6585	<0.001†
Yes	775	11225 (11181)	1550	7041 (9520)	4183	<0.001†

Abbreviations: HAI, healthcare-associated infection; SD, standard deviation; MSSA, methicillin-susceptible *S. aureus*; MRSA, methicillin- resistant *S. aureus*.

* Any of the 7 classes of severe illnesses (cancer, dialysis-dependent end stage renal disease, liver cirrhosis with complications, chronic ventilator dependence, generalized autoimmune syndrome, spinal injury/myeleterosis, and major trauma).

‡ At an exchange rate of 30 New Taiwan Dollars (NT$s)/US$.

† Statistically significant, after Bonferroni correction (*P*<0.05/20 = 0.0025).

## Discussion

This study is the largest cohort study to date that has investigated the negative effects of *S. aureus* HAIs on the outcomes of hospitalized patients. Using national databases, we included 3070 inpatients with *S. aureus* HAIs and 6140 matched uninfected inpatients. Our results show that *S. aureus* HAIs significantly increased the risks for long-term mortality and disabilities including new-onset chronic ventilator dependence and new-onset dialysis-dependent end-stage renal disease, with an excess one-year risk of 20.2%, 7.3%, and 2.6%, respectively (all *P*s<0.001). *S. aureus* HAIs were also associated with an excess hospital stay of 12 days and an extra hospital cost of $5978 (*P*s<0.001).

In addition to a large sample size, our study has the advantage of enhancing comparability by individually matching the *S. aureus* HAI cases to uninfected inpatients on potential confounding factors including age, gender, hospital, primary specialty/subspecialty, and underlying disease severity. Analysis of the validation variables did show a lack of difference in most baseline characteristics (e.g. the frequency of cardiovascular diseases, elective surgery, and antineoplastic agent use), with the exception of a slightly higher proportion of patients with diabetes mellitus and hypertension in the uninfected group. The most likely explanation for the difference is that diabetes mellitus and hypertension were more likely to be recorded among the five major diagnoses of the patient in the NHI database for the uninfected group that had a lower average number of diagnoses. Even if the result reflects a genuine difference in these two comorbidities, the higher proportions of patients with diabetes mellitus and hypertension (which may adversely affect the outcomes) in the uninfected group would have caused an underestimation for the negative impact of *S. aureus* HAIs and thus the actual excess risks would have been higher than the observed values.

Our findings on the excess mortality, prolonged hospital stay, and extra hospital costs associated with *S. aureus* HAIs are consistent with the existing literature [15]–[20]. Previous studies, which involved smaller numbers of patients and mainly focused on surgical site infections, reported an excess 90-day crude mortality of 10.5–16.8% for patients with *S. aureus* surgical site infections [15], [17], [18]. Using population-based data, our study validates the previous results and found an excess one-year mortality of 12.4%. Furthermore, our study extends the results to patients with *S. aureus* HAIs in general. We also first show that patients with *S. aureus* pneumonia and bloodstream infection suffered the highest excess one-year mortality of 28.5% and 21.3%, respectively.

In addition to an excess infection-related mortality, our study shows that *S. aureus* HAIs increase the risk for long-term disability. Severe *S. aureus* infections can cause acute organ dysfunction [26], particularly in patients with pre-existing chronic lung or renal disease(s). Blot et al. compared 85 cases of *S. aureus* bacteremia with 170 matched uninfected patients and found that the former had a significantly longer length of ventilator dependence than the latter [3]. Reach et al. composed a large study of 1575 matched pairs and found that MRSA patients were more likely to undergo mechanical ventilation than uninfected patients (excess risk: 7.5%) [27]. Our study first provides evidence on the potential irreversibility of *S. aureus* HAIs-related ventilator dependence and renal failure, showing that *S. aureus* HAIs increased the risks for new-onset chronic ventilator dependence and dialysis-dependent end-stage renal disease by 7.3% and 2.6%, respectively, compared with patients with the same type and severity of underlying disease but without HAIs. Therefore, *S. aureus* HAIs can cause irreversible organ dysfunction and profoundly affect the patient’s long-term well-being.

The excess risks for long-term mortality and disability highlight the importance to reduce occurrence of *S. aureus* HAI, which is a preventable disease. One of the main causes for the spread of MRSA within hospitals is poor hand hygiene compliance among healthcare workers [28]. Studies have found that the incidence of HAIs can be decreased by the introduction of hand hygiene programs and other measures [29]. There is growing literature supporting the beneficial effects of hand hygiene [30], [31]. A systemic review of 30 intervention studies suggested that 10–70% of HAIs are probably preventable with appropriate infection control [32]. A recent randomized controlled trial proves that active surveillance and decolonization of nasal *S. aureus* carriers on admission can further reduce the incidence rate of surgical site infection [33].

Our results on the excess mortality/disability and excess hospital stay/costs indicate that a reduction in incidence of *S. aureus* HAIs can translate to improved long-term outcomes and significant cost savings, particularly when the huge financial burdens of providing long-term ventilator and dialysis services are taken into consideration.

Our study was limited by the voluntary nature of TNIS participation and HAI case notification. The 114 hospitals in current study may not represent all hospitals in Taiwan. Nevertheless, we minimize the potential effect of self-selection bias on the estimated excess risk associated with *S. aureus* HAIs, by individually matching the *S. aureus* HAI cases to uninfected inpatients by the hospital. Because the notification of HAI cases was also voluntary, it is possible that some of the 6140 matched uninfected inpatients might indeed have HAIs, which would have caused an underestimation of *S. aureus* HAI-associated excess risks for long-term mortality and disability. Therefore, our findings represent a conservative estimate for the negative impact of *S. aureus* HAIs.

### Conclusion

*S. aureus* HAIs have substantial negative effect on the long-term outcome of hospitalized patients in terms of both mortality and disability, which should be taken into consideration in future cost-effectiveness studies of the control and prevention interventions for *S. aureus* HAIs.
